# Long non-coding RNA RP11-59H7.3 promotes cell proliferation and invasion metastasis in colorectal cancer by miR-139-5p/NOTCH1 axis

**DOI:** 10.18632/aging.103331

**Published:** 2020-06-06

**Authors:** Xiaojian Zhu, Chen Luo, Fanqin Bu, Kang Lin, Zhengming Zhu

**Affiliations:** 1 Gastrointestinal Surgery, The Second Affiliated Hospital of Nanchang University, Nanchang, China; 2 Jiangxi Medical College of Nanchang University, Nanchang, China; 3 Jiangxi Province Key Laboratory of Molecular Medicine, Nanchang, China

**Keywords:** LncRNA, RP11-59H7.3, MiR-139-5p, NOTCH1, colorectal cancer

## Abstract

Increasing evidence suggests long non-coding RNAs (lncRNAs) are distinctively expressed in several cancers. However, the functions of these lncRNAs in cancer development remain unknown. In the current study, we report high expression of a novel lncRNA, RP11-59H7.3, and its association with prognosis in colorectal cancer (CRC) patients. Functional analyses of this lncRNA revealed its role in promoting proliferation and progression of the cell cycle, as well as enhancement of cell migration and invasion. Furthermore, our results revealed that knockdown of RP11-59H7.3 promoted cell apoptosis, with luciferase reporter assays showing that it directly binds to miR-139-5p. Knockdown of this lncRNA significantly reduced expression of NOTCH1, a direct target of miR-139-5p. Additionally, we show that suppression NOTCH1 by miR-139-5p could be partially rescued by overexpressing RP11-59H7.3. Analysis of the relationship between RP11-59H7.3 and miR-139-5p, in CRC tissues, showed a negative correlation while a positive association was observed between the RP11-59H7.3 expression and levels of NOTCH1. Taken together, these results demonstrated that the RP11-59H7.3/miR-139-5p/NOTCH1 axis functions as a key regulator in CRC metastasis. RP11-59H7.3 represents a potential biomarker for CRC diagnosis and could be an important target for development of novel therapies to manage the disease.

## INTRODUCTION

Colorectal cancer (CRC) is one of the most common malignancies, accounting for an estimated 1.1 (6.1%) million new cancer cases and 0.88 (9.2%) million cancer-related deaths per year worldwide [1]. Despite great progress in CRC screening and development of therapies against the disease, the 5-year survival rate among CRC patients remains unsatisfactory [2], mainly due to its high metastatic nature. Consequently, approximately 90% of CRC-related deaths occur as a result of metastasis [3]. Unraveling the molecular mechanisms underlying CRC metastasis is critical to development of early intervention strategies, especially for individuals with high risk of metastasis.

Long noncoding ribonucleic acids (LncRNAs) are 200 nucleotide long molecular transcripts that are not translated into proteins [4]. Previously, lncRNAs were regarded as “trash RNA” although this did not raise attention. Recently, several lines of evidence have shown that these RNAs play crucial roles in physiological processes of several tumors, including cell cycle distribution [5, 6], growth [7, 8], and metastasis [9]. Additionally, many studies have revealed dysregulation of several lncRNAs in CRC, especially SLCO4A1-AS1, SNHG6, HOXD-AS1, and NEAT1. lncRNAs function as tumor suppressors or oncogenes in progression and tumorigenesis of CRC, depending on the circumstances. For example, SLCO4A1-AS1 enhances proliferation of CRC cells by increasing autophagy through miR-508-3p/PARD3 axis [10]. In addition, lower expression levels of nuclear HOXD-AS1 inhibit colorectal tumor progression by suppressing HOXD3-induced integrin*β*-3 transcriptional activating and signaling of MAPK/AKT [11]. Similarly, Zhang et al. reported that upregulation of NEAT1 in CRC enhances disease progression through miR-193a-3p sponging. Additionally, NEAT1 stimulates signaling of Wnt/β-catenin and enhances CRC progression through a synergistic interaction with DDX5 [12, 13]. These findings indicate that several lncRNAs are individually expressed in CRC, participate in initiation and progression of CRC by competitively binding onto miRNAs and influence expression of several target molecules. It is, therefore, critical to unravel the specific lncRNAs associated with CRC to improve personalized therapy for patients.

In the current study, we observed lncRNA RP11-59H7.3 overexpression in colorectal tumor tissues, cells, and serum, and this expression was strictly associated with poor prognosis. Functional analyses revealed that this factor could promote growth of colorectal cancer cells and migration by inhibiting apoptosis. Additionally, deregulation of the lncRNA revealed that it could inhibit cell growth and migration in CRC orthotopic xenografts. However, we observed similar findings when lncRNA RP11-59H7.3 was upregulated. Previous studies have shown that RP11-59H7.3 enhances cancer pathogenesis by functioning as miR-139-5p competitive endogenous RNA (ceRNA), a vital cancer-inhibiting microRNA (miRNA). miR-139-5p plays a crucial role in tumor formation and progression in humans, including colorectal cancer [14–16]. Our results revealed a unique pathway for RP11-59H7.3/ NOTCH1/miR-139-5p regulation in colorectal cancer, suggesting that RP11-59H7.3 is a new biomarker for prognosis. This factor is expected to provide individualized diagnosis and aid in designing novel therapeutic options for management of postoperative colorectal cancer.

## RESULTS

### RP11-59H7.3 is upregulated in human CRC tissues, serum and cells

**Figure 1:**
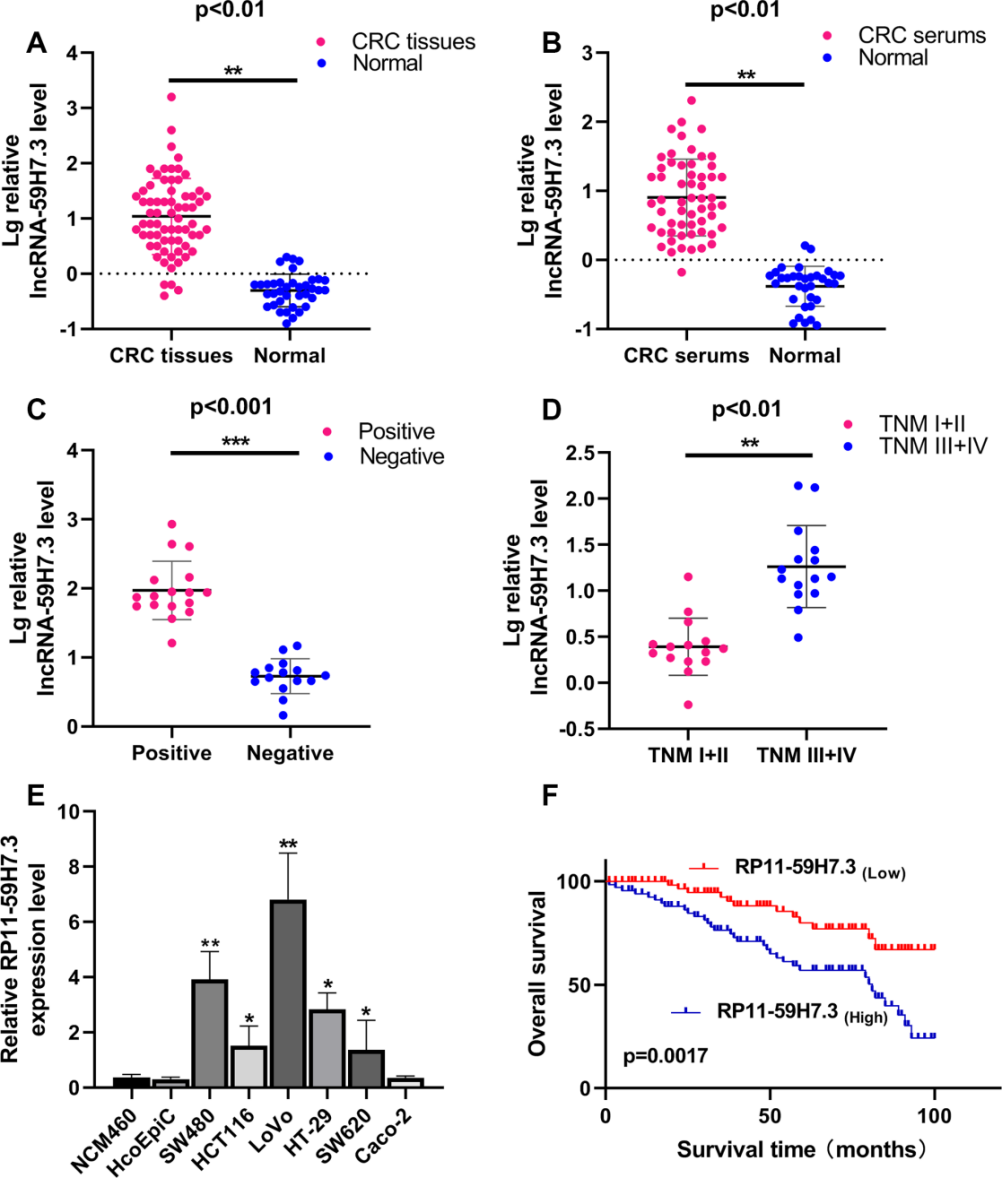
**RP11-59H7.3 is upregulated in tumor tissues, serums and cell lines of CRC.** (**A**) Relative expression levels of RP11-59H7.3 in 68 paired CRC and paired adjacent healthy tissues were quantified by RT-qPCR. (**B**) Relative expression levels of RP11-59H7.3 in 57 CRC serums and negative control sera were quantified by RT-qPCR. (**C**, **D**) Relative RP11-59H7.3 expression in the CRC patients for lymph node positive, lymph node negative and stage I+II, stage III+IV. (**E**) Relative RP11-59H7.3 expression in CRC cell lines (SW480, HCT116, LoVo, HT29, SW620, Caco-2) compared to normal colonic epithelial cell line NCM460 and HcoEpic. (**F**) Kaplan-Meier survival analysis of the overall survival in two groups defined by low and high expression of RP11-59H7.3 in patients with CRC. ***p < 0.001; ** *p* < 0.01; *p < 0.05.

To investigate expression profiles of RP11-59H7.3 in CRC, we first measured the mRNA levels of the lncRNA in 68 pairs of CRC and paired adjacent healthy tissues using RT-qPCR. We found significantly (*p* < 0.01) elevated levels of the lncRNA in tumor tissues (28.51 ± 48.11) compared with controls (0.96 ± 2.76) (Figure 1A). Similarly, we found elevated RP11-59H7.3 levels in 57 pairs of CRC serum specimens (Figure 1B, *p* < 0.01). Additionally, the expression levels of this lncRNA was significantly (*p* = 0.0018) higher in stage III +IV than stage I+II patients (Figure 1D,) and was closely correlated (*p* = 0.033) with lymphatic metastasis (Figure 1C).

Next, we analyzed the relationship between levels of RP11-59H7.3 expression and clinical-pathological parameters in CRC patients. An outline of the clinicopathologic data is presented in Table 1. We found a correlation between high RP11-59H7.3 levels and lymph node metastasis status (*p = 0.033) and advanced TNM stage (**p = 0.002) (Table 1). However, the high expression did not have a significant association with other clinical features, including gender, age at diagnosis, differentiation, depth of invasion in our study.

**Table 1:** The correlation of the expression of RP11-59H7.3 with clinical features in colorectal cancer.

**Characteristics**	**Number of case**	**RP11-59H7.3 expression**		***p* value**
		**High (n=29)**	**Low (n=29)**	
Gender				*p* = 0.791
Male	25(43.1%)	12	13	
Famale	33(56.9%)	17	16	
Age at diagnosis				*p* = 0.517
<60	12(20.7%)	7	5	
≥60	46(79.3)	22	24	
Differentiation				*p* = 0.424
poor	4(6.9%)	3	1	
Moderately	42(72.4%)	19	23	
well	12(20.7%)	7	5	
Depth of invasion				*p* = 0.421
T1,T2	23(39.7%)	10	13	
T3,T4	35(60.3%)	19	16	
Location				*p* = 0.297
Transverse colon	3(5.2%)	1	2	
Ascending colon	16(27.6%)	9	7	
Descending colon	17(29.3%)	11	6	
Sigmoid colon	22(37.9%)	8	14	
Lymph node status				*p* = *0.033
Positive	24(41.4%)	16	8	
Negative	34(58.6%)	13	21	
TNM stage				*p* = **0.002
I+II	26(44.8%)	7	19	
III+IV	32(55.2%)	22	10	

** *p* < 0.01; *p < 0.05.

**Figure 2:**
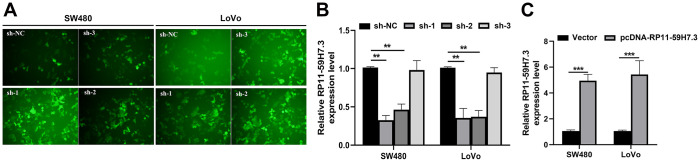
**Overexpression and stable knockdown of RP11-59H7.3 in CRC cells.** (**A**) Representative images of SW480 and LoVo cells transfected with sh-1, sh-2, sh-3, or sh-NC. (**B**, **C**) Validation of knockdown and overexpression efficacy of RP11-59H7.3 in CRC cell lines by RT-qPCR. ** *p* < 0.01; * *p* < 0.05.

Then, we detected the expression levels of RP11-59H7.3 in the CRC cell lines using RT-qPCR. We found significantly higher (*p* < 0.05) levels of RP11-59H7.3 in tissues of CRC cell lines relative to those from normal human colon epithelium cell line NCM460 and HcoEpic (Figure 1E). Notably, expression levels of RP11-59H7.3 were considerably higher in LoVo and SW480 CRC cell lines, whereas HT29, HCT116, and SW620 cells expressed relatively lower levels of RP11-59H7.3. For this reason, we selected LoVo and SW480 cells for further experiments. To avoid off-targeting by the transcripts, we designed three candidate shRNAs, and sh-1 and sh-2 that had optimized interference efficiency (Figure 2A, *p* < 0.01). Relative RP11-59H7.3 expression in LoVo and SW480 after knockdown or overexpression was detected by RT-qPCR (Figure 2B, 2C).

### High RP11-59H7.3 expression in CRC is associated with poor prognosis

Estimates from the Kaplan–Meier survival curves indicated significantly shorter disease-free survival (DFS) and overall survival (OS) in patients having high levels of RP11-59H7.3 compared to those with low levels in cancer cells 43.326 ± 4.247 vs. 69.415 ± 3.521 months; log rank=9.179, *p=*0.0017). (Figure 1F). Multivariate and univariate analyses indicated that advanced TNM stage and overexpression of RP11-59H7.3 were significantly associated to unfavorable overall survival, suggesting that RP11-59H7.3 is an independent predictive factor for poor disease outcomes (95% CI: 1.105-5.731; HR = 2.015; *p* = 0.029) (Table 2. These findings indicate the potential for RP11-59H7.3 as a risk factor and prognostic predictor in patients with colorectal cancer).

**Table 2:** Multivariate analysis of clinicopathological factors for disease-specific survival.

**Variable**	**Subset**	**Univariate analysis**		**Multivariate analysis**	
		***p*-value**	**HR (95% CI)**	***p*-value**	**HR (95% CI)**
Gender	Male/female	0.634	0.667 (0.457–1.649)	--	--
Age at diagnosis(years)	<60/≥60	0.879	0.721 (0.558–1.845)	--	--
Differentiation	Well+moderately/poorly	0.319	1.355 (0.683–2.691)	--	--
Depth of invasion	T1+T2/T3+T4	*0.037	1.116 (0.522–4.238)	0.074	3.463 (0.647–6.033)
Location	Colon/rectum	0.713	0.781 (0.352–1.773)	--	--
Lymph node status	Positive/Negative	*0.015	2.571 (1.306–4.204)	0.216	2.154(1.146–6.915)
TNM stage	I+II/III+IV	*0.013	12.113 (5.044–32.352)	**0.004	7.306(2.231–33.013)
RP11-59H7.3	High/low	**0.009	3.545 (1.453–8.047)	*0.029	2.015 (1.105–5.731)

** *p* < 0.01; *p < 0.05.

### RP11-59H7.3 enhances cell cycle progression, cell proliferation, and suppresses apoptosis of CRC cells

**Figure 3:**
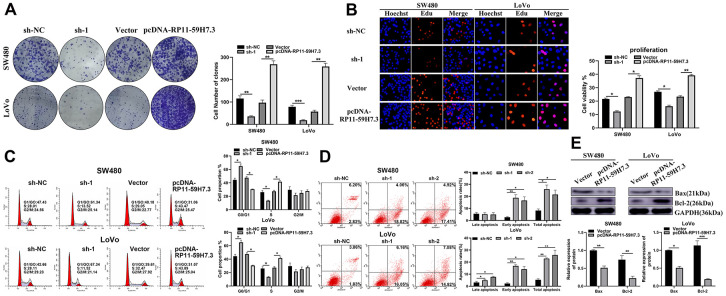
**RP11-59H7.3 promotes proliferation, cycle progression and inhibits apoptosis of CRC cells in vitro.** (**A**) effects of overexpression and knockdown of RP11-59H7.3 on the formation of colonies in colorectal cancer cells. (**B**) the Edu proliferation assays were applied to evaluate the CRC cell viability prior transfected with pcDNA-RP11-59H7.3 compared to vector control. (**C**) cell cycle analysis was conducted in both LoVo and SW480 cells prior transfected with sh-1 and sh-NC or vector and pcDNA-RP11-59H7.3. (**D**) programmed cell death analysis was conducted in sh-NC, sh-1, and sh-2 transfected cell lines. (**E**) western blot was conducted to analyze the apoptosis-related proteins expression in colorectal cancer cells. ***p < 0.001; ** *p* < 0.01; * *p* < 0.05.

The Edu proliferation assay revealed a marked increase in cell proliferation in SW480 and LoVo cells after transfection with pcDNA-RP11-59H7.3 compared to respective control groups. Additionally, RP11-59H7.3 knockdown significantly (*p* < 0.05) inhibited CRC cell proliferation (Figure 3B). According to colony formation tests, the rate of formation of colonies was low (*p* < 0.01) when RP11-59H7.3 was inhibited in CRC cells, but its upregulation resulted in high colony numbers (Figure 3A). These findings revealed that RP11-59H7.3 contribute to the growth of CRC cells.

The growth-potentiating effects of RP11-59H7.3 in CRC were further evaluated in SW480 and LoVo cells by analyzing cell cycle progression using flow cytometry. The results showed that overexpression of RP11-59H7.3 resulted in increased (in the S phase), but reduced the number of cells in G1 phase. S further validation of the results was performed for both SW480 and LoVo (Figure 3C), with the results suggesting that the biological functions of RP11-59H7.3 contributed to promoted cell cycle progression in CRC.

We also analyzed programmed cell death in CRC by applying annexin V-FITC/PI staining on sh-RP11-59H7.3-transfected LoVo and SW480 cells via flow cytometry. We found that higher percentages of LoVo (25.91% ± 0.51%) and SW480 (RP11-59H7.3 knockdown) [17.23% ± 2.57%] underwent apoptosis, compared to NC controls (16.56% ± 1.35 in SW480 cells, 18.54% ± 1.68% in LoVo cells, *p* < 0.05) (Figure 3D). Similarly, analysis of protein expression in apoptotic cell lines, showed that silencing of RP11-59H7.3 significantly reduced (*p* < 0.05) Bcl-2 expression and enhanced levels of Bax, relative to NC cells (Figure 3E).

### RP11-59H7.3 promotes CRC cell migration and invasion *in vitro*

**Figure 4:**
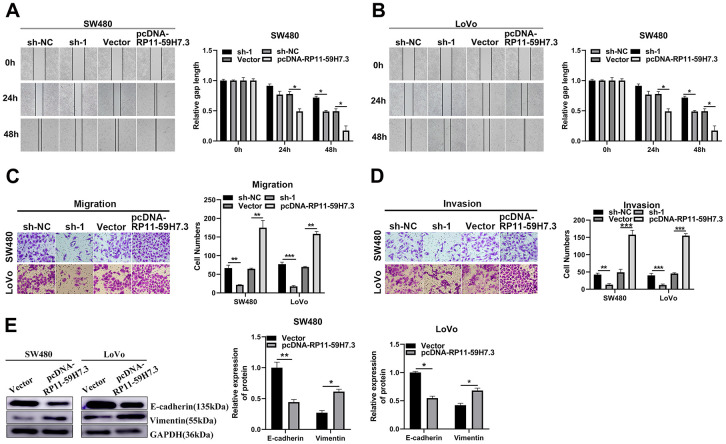
**RP11-59H7.3 enhances cell movement and invasion in colorectal cancer cells.** (**A**, **B**) wound-healing assay was performed to determine the horizontal migration ability of CRC cells using overexpression or knockdown of RP11-59H7.3 in CRC cells, and relative gap length calculations were performed and a histogram was plotted. (**C**, **D**) representative bar graphs and images that depicts the ability of CRC cells with silenced or overexpressed RP11-59H7.3 to migrate and invade neighboring cells. (**E**) western blot was performed to evaluate the metastasis-related protein with RP11-59H7.3 overexpression or silenced expression in CRC cells. Data from western blot analysis is represented as a quantification graph normalized to the GAPDH levels and the statistical tests. ***p < 0.001; **p < 0.01*; *p* < 0.05.

To determine whether RP11-59H7.3 contributes to CRC metastasis, we analyzed its effect on migration and invasion abilities, and found that SW480 and LoVo cells transfected with pcDNA-RP11-59H7.3 displayed a notably faster recovery compared to controls. Conversely, CRC cells, in which RP11-59H7.3 had been knocked down, showed a slower recovery than controls (Figure 4A and 4B). Transwell assays, performed to measure the impact of RP11-59H7.3 on CRC metastasis, showed that overexpression of RP11-59H7.3 significantly (*p* < 0.01) facilitated migration and invasion in both SW480 and LoVo cells, whereas its knockdown suppressed these processes in CRC cells (Figure 4C, 4D).

We also analyzed expression profiles of proteins that regulate cell metastasis, targeted by RP11-59H7.3, by examining the levels of tumor-suppressor-related proteins in pcDNA-RP11-59H7.3 CRC cells. We found downregulation in expression of vimentin, an essential protein related to tumor metastasis, and a marked elevation of E-cadherin in pcDNA-RP11-59H7.3 CRC cells (*p* < 0.05) (Figure 4E). This indicated that alteration in levels of these proteins plays a role in pcDNA-RP11-59H7.3-mediated malignant progression. Reverse profiles in protein expression were observed in CRC cells when RP11-59H7.3 was overexpressed using sh-RP11-59H7.3 (*p* < 0.05) (Figure 4E).

### RP11-59H7.3 sponges miR-139-5p

**Figure 5:**
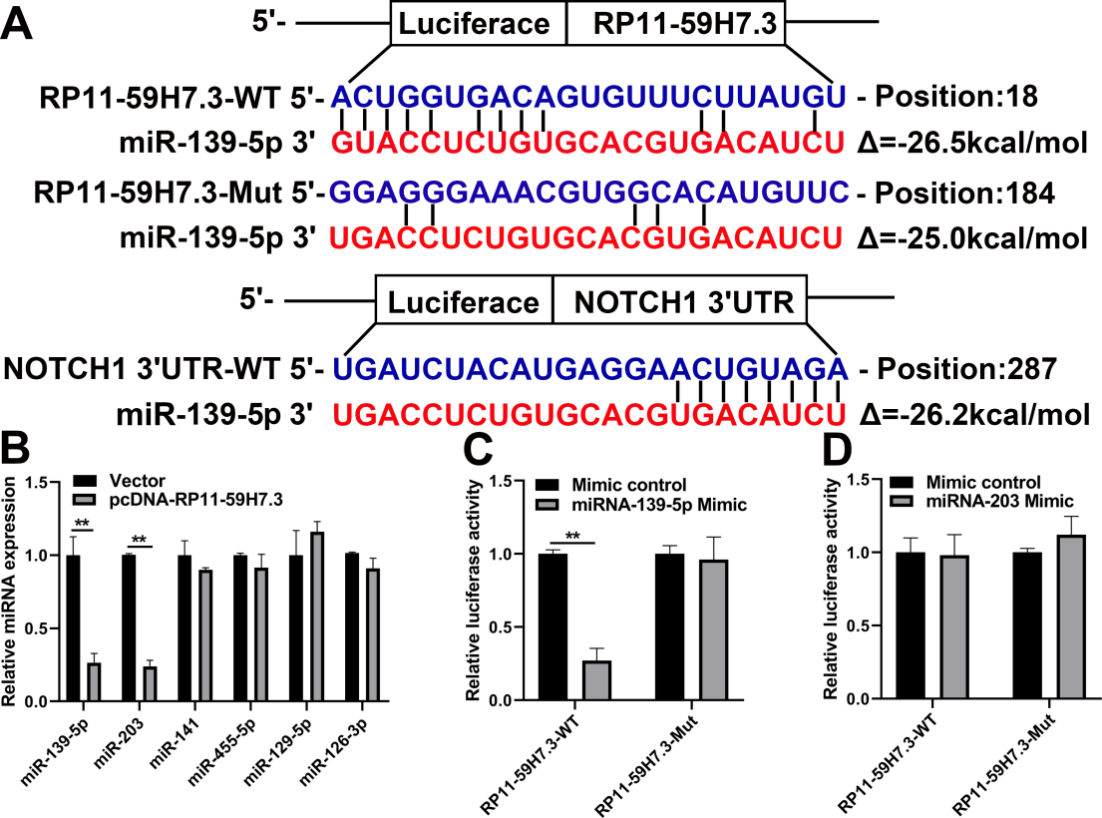
**miR-139-5p is directly targeted by RP11-59H7.3.** (**A**) miR-139-5p-binding sequence in NOTCH1 3'UTR and RP11-59H7.3. The mutation was induced in RP11-59H7.3 in the site complementary to the miR-139-5p binding. (**B**) RT-qPCR was performed to determine levels of miRNA in cells of SW480 post-transfection by pcDNA-RP11-59H7.3. The activity of luciferase in cells of 293T co-transfected by miR-139-5p (**C**) or miR-203 (**D**) mimics and the luciferase reporters (mutant RP11-59H7.3) or control (wild type RP11-59H7.3). The activity of renilla luciferase was determined then normalized to firefly luciferase activity level. Each experiment was repeated thrice, and the data are summarized as mean ± SD (two-tailed Student’s t-test). **p < 0.01; *p < 0.05.

To investigate the mechanisms of RP11-59H7.3 action in development of colorectal cancer, we used miRanda and TargetScan to detect miRNAs that could attach to RP11-59H7.3. Analysis revealed RP11-59H7.3 binding sites as well as potential miRNAs targets, including miR-139-5p, -203, -141, -455-5p, -129-5p, and -126-3p (Figure 5B). We also performed a RT-qPCR analysis of the aforementioned miRNAs in CRC-LoVo cells post-transfection by pcDNA-RP11-59H7.3 and noted significantly (*p* < 0.001) elevated miR-203 and miR-139-5p expression following overexpressing of RP11-59H7.3. On the other hand, no change in the expression levels of the other three miRNAs was observed (Figure 5B). When the miR-139-5p mimic was introduced, we found a significant reduction in luciferase activity in the WT-RP11-59H7.3 relative to the negative control. However, luciferase activity in the Mut-RP11-59H7.3 was not affected (*p* < 0.01) (Figure 5C). Conversely, no inhibitory effect of miR-203 on luciferase activity was observed in the wt-RP11-59H7.3, relative to the negative control (Figure 5D).

**Figure 6:**
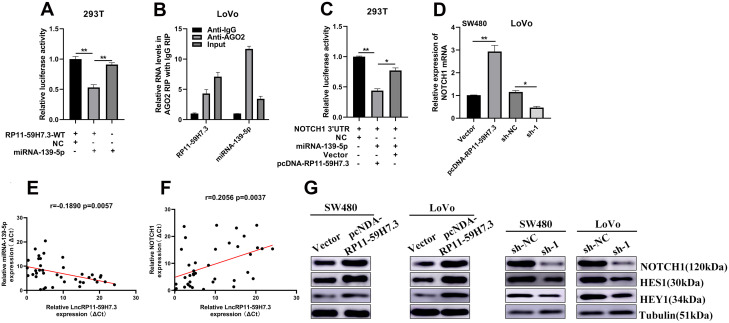
**RP11-59H7.3 sponges miR-139-5p and moderate expression of NOTCH1.** (**A**) The activity of luciferase of a luciferase reporter vector (pLuc) harboring mutant RP11-59H7.3 or wild-type co-transfected by miR-139-5p was evaluated using the dual luciferase assay. (**B**) cell lysates of SW480 cells were used for RIP with an IgG antibody or anti-Ago2 antibody. The miR-139-5p and RP11-59H7.3 levels were analyzed using RT-qPCR. (**C**) pLuc plasmid and miR-139-5p harboring NOTCH1 3'UTRs were co-transfected by pcDNA-RP11-59H7.3 or empty vector into cells of 293T to determine whether RP11-59H7.3 could act as a ceRNA of miR-139-5p. (**D**, **G**) The NOTCH1 expression levels in cells of SW480 prior transfected by pcDNA-RP11-59H7.3 and LoVo cells prior transfected by sh-lncRNA-RP11-59H7.3 were determined using western blot and RT-qPCR. (**E**, **F**) Analysis of correlation between expression of RP11-59H7.3/miR-139-5p and RP11-59H7.3/NOTCH1. **p < 0.01; *p < 0.05.

We then evaluated the underlying relationship between miR-139-5p with RP11-59H7.3 using luciferase reporter assays. We observed that miR-139-5p overexpression led to a marked inhibition of the reporter activity of pcDNA-RP11-59H7.3-WT (Figure 6A), suggesting sequence-specific binding and inhibition of RP11-59H7.3 by miR-139-5p. To further verify that RP11-59H7.3 binds to miR-139-5p, an RNA Immunoprecipitation (RIP) assay using an anti-Ago2 antibody was performed. Data showed that both RP11-59H7.3 and miR-139-5p were markedly enriched in Ago2 complex, indicating that RP11-59H7.3 is included in miRNPs, probably through binding with miR-139-5p (Figure 6B).

### RP11-59H7.3 modulates NOTCH1 expression by competitively binding miR-139-5p

Previous studies have shown that miR-139-5p inhibits CRC tumorigenesis, development, and chemoresistance by regulating NOTCH1 expression. To ascertain whether these effects could be regulated by RP11-59H7.3 on the miR-139-5p/NOTCH1 pathway, we first evaluated the relationship among RP11-59H7.3, miR-139-5p and NOTCH1 using luciferase assays. We found that overexpression of RP11-59H7.3 but not the vector control, blocked the inhibitory effect of miR-139-5p on relative luciferase expression of pLuc-NOTCH1-3'UTR (Figure 6C). These results confirmed that RP11-59H7.3 abolishes miR-139-5p-mediated repressive activity on NOTCH1 by competitively binding miR-139-5p. In addition, knocking down RP11-59H7.3 significantly reduced the endogenous NOTCH1 expression in CRC cells (Figure 6C). In contrast, NOTCH1 expression resulted in a marked increase in RP11-59H7.3 overexpressing CRC cells (Figure 6D). Additionally, a positive relationship was observed between levels of NOTCH1 and RP11-59H7.3 in CRC tissues (Figure 6F). Conversely, we found a negative correlation between lncRNA expression of RP11-59H7.3 and miR-139-5p in CRC tissues (Figure 6E). Taken together, these results demonstrate that RP11-59H7.3 can regulate NOTCH1 activity by sponging miR-139-5p in both CRC cell lines and clinical CRC tumors.

### RP11-59H7.3 triggers tumor-enhancing function in CRC by regulating miR-139-5p/NOTCH1 axis

**Figure 7:**
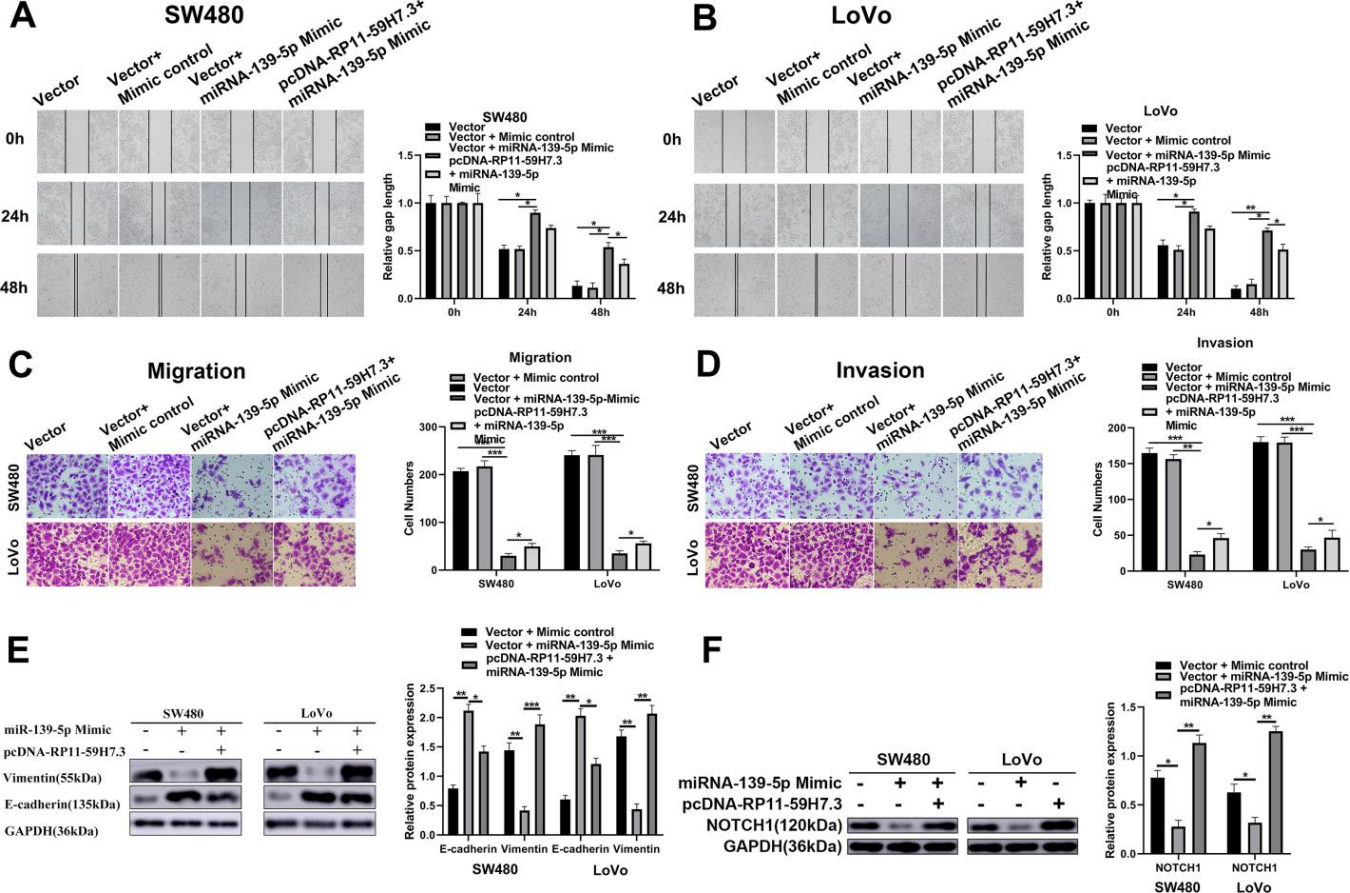
**RP11-59H7.3 promotes CRC cell migration and invasion through miR-139-5p/NOTCH1 axis.** (**A**, **B**) wound-healing assays were performed in LoVo and SW480 cells prior transfected by miR-139-5p mimic or co-transfected by pcDNA-RP11-59H7.3 and miR-139-5p mimic, and calculations of relative gap distance were completed and a histogram plotted. (**C**, **D**) cell movement and invasion assays were applied to determine the invasion ability and vertical migration of colorectal cancer cells, and the cell number was calculated and represented on a histogram. (**E**) Western blot was performed to determine the metastasis-related protein expression in colorectal cancer cells. (**F**) Relative NOTCH1 protein levels in colorectal cells after RP11-59H7.3 overexpression and Mimics of miR-139-5p. **p < 0.01; *p < 0.05.

Previous studies have implicated miR-139-5p in the regulation of apoptosis, cell proliferation, suppression of tumor invasion, and cell migration by regulating NOTCH1 in colorectal cancer. To determine whether RP11-59H7.3 triggers tumor-enhancing roles in CRC by controlling the miR-139-5p/NOTCH1 axis, we examined the effects of NOTCH1 and miR-139-5p on RP11-59H7.3-induced cell invasion and movement. Results showed that overexpression of miR-139-5p or silencing of NOTCH1 inhibited RP11-59H7.3-trigged migration and invasion in CRC cells (Figure 7A). Additionally, over-expressing miR-139-5p or silencing NOTCH1 restored cell migration in CRC cells overexpressing RP11-59H7.3 (Figure 7D). In general, these findings indicated that RP11-59H7.3 triggers tumor-enhancing functions in colorectal cancer cells to some extent, and this is through miR-139-5p sponging and NOTCH1 regulation.

### RP11-59H7.3 knockdown inhibited cell proliferation and metastasis in CRC orthotopic xenografts

**Figure 8:**
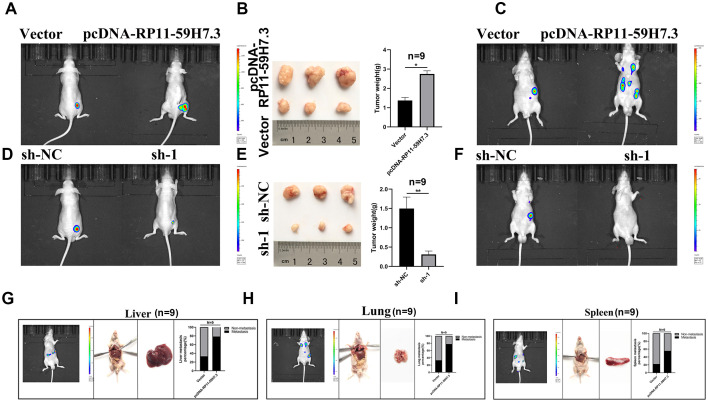
**The lncRNA RP11-59H7.3 deregulation inhibits cell invasion and proliferation in CRC orthotopic xenografts.** (**A**–**C**) representative IVIS images showing size of tumors (**A**), macroscopic appearance (**B**), and metastasis (**C**) in sh-lncRNA RP11-59H7.3 groups vs control groups. (**D**–**F**) Representative IVIS images showing tumor size (**D**), macroscopic appearance (**E**), and metastasis (**F**) in pcDNA-RP11-59H7.3 groups vs control groups. (**G**–**I**) Representative macroscopic appearance and IVIS image of metastatic foci (white arrows) in the liver (**G**), lung (**H**), and spleen (**I**). **p < 0.01; *p < 0.05.

Data from in vitro experiments indicated that RP11-59H7.3 promotes proliferation and invasion of CRC cells. In vivo experiments, we also used orthotopic xenograft mouse models to detect the anti-tumorigenic role of shlncRNA RP11-59H7.3. First, we labeled SW480 cells with luciferase expression and transfected them with RP11-59H7.3-shRNA and functional RP11-59H7.3-cDNA. Then, we inoculated the transfected cells into the left renal capsule of nude mice, and monitored tumor size and metastasis through an In Vivo Imaging System (IVIS). After 6 weeks, IVIS results revealed a reduction in expression of tumor luciferase in the shlncRNA RP11-59H7.3 group, relative to the shRNA controls (Figure 8D). Meanwhile, the results also showed that deregulation of RP11-59H7.3 effectively suppressed tumor size and metastasis (Figure 8E, 8F). Conversely, overexpression of lncRNA RP11-59H7.3 increased tumor size and metastasis compared to the control group (Figure 8A–8C), while overexpression of lncRNA RP11-59H7.3 promoted metastasis in the lung, liver, spleen and diaphragm (Figure 8G–8I). Taken together, the results summarized in Figure 8A–8I demonstrated that shlncRNA RP11-59H7.3 functions as a tumor suppressor by inhibiting tumorigenesis and metastasis in CRC cells.

## DISCUSSION

In the current study, we screened the TGCA database and found considerable overexpression of RP11-59H7.3, a unique lncRNA related to colorectal cancer (CRC) in tissues of patients. Initial analysis showed significant overexpression of the lncRNA in tumor tissues, with similar findings later confirmed in cells and serum. Further analyis associated its expression with tumor stages and poor prognosis and later implicated it in enhanced proliferation, migration, cell division process and apoptosis in CRC. Moreover, *in vivo* tumor growth experiments verified the effect of overexpression of RP11-59H7.3 on tumor progression. Our results also revealed that RP11-59H7.3 deregulation effectively inhibited the growth and migration of the tumor. Conversely, oelncRNA RP11-59H7.3 enhanced tumor growth and spread to other tissues, when compared to the mock group and was also observed to promote the spread of cancer cells in the liver, lung, diaphragm, and spleen. Analysis involving shlncRNA RP11-59H7.3 indicated that this factor acts as a cancer inhibitor, by suppressing tumor growth and migration in colorectal cancer cells. Additionally, the results herein further revealed that RP11-59H7.3 performs tumor-enhancing roles through miR-139-5p sponging and regulation of NOTCH1 expression in colorectal cancer.

In recent past, the functions of lncRNAs in pathogenesis of human diseases, particularly in cancer, have received numerous attention [17, 18]. Currently, researchers have hypothesized that lncRNAs play a significant role in cancer formation and progression and are therefore targeted during development of new cancer therapies [19]. Several studies have revealed differential expression of cancer-specific factors in the cell. These factors specifically bind and regulate expression of lncRNAs, thereby promoting tumor growth. Numerous studies have reported regulation of colorectal cancer cells by lncRNA (through targeting of mRNAs or microRNAs). For instance, LINC00152, a lncRNA that enhances proliferation and migration of tumor cells has been found to confer resistance to 5-FU in CRC by suppressing miR-139-5p [15]. Similarly, significant upregulation of LINC02418 was reported in CRC cells, and LINC02418-miR-1273g-3p-MELK axis found to perform a crucial function in tumorigenesis of CRC [20]. Additionally, lncRNA MFI2-AS1 has been found to enhance CRC cell growth, metastasis, and infiltration via the miR-574-5p/MYCBP axis [21]. Based on these reports, identification of molecular regulatory networks between lncRNA/miRNA and genes could provide more targets for novel therapies for the management of colorectal cancer.

Previous studies have shown that miRNA-139-5p suppresses cell growth and infiltration in CRC by inhibiting expression of NOTCH1 [15, 16, 22]. However, of the roles played by lncRNAs in miRNA-139-5p-NOTCH1-associated tumorigenesis have not been elucidated. According to a previous study, lncRNAs candidates were identified using a human lncRNA target prediction tool (DIANA TOOLS), with the results indicating overexpression of lncRNA RP11-59H7.3 in colorectal cancer relative to adjacent healthy renal tissues. Moreover, CRC patients who were in the high lncRNA RP11-59H7.3 overexpression group showed worse disease outcomes, compared to those who were in the low lncRNA RP11-59H7.3 expression group based on Kaplan-Meier survival estimates.

The role of lncRNAs in sponging miRNA and control of its expression levels has also been extensively studied [18, 23]. Particularly, lncRNAs sponge miRNAs via MREs and prevent downstream repression of mRNAs. For instance, BC032469 was found to enhance expression of HTERT, thereby boosting tumor proliferation, in gastric cancer, through miR-1207-5p sponging [24]. With regards to chronic myeloid leukemia, a lncRNA, BGL3, was found to act as a ceRNA and cross-control PTEN expression by sponging six miRNAs (miR-106a, miR-93, miR-106b, miR-20a, miR-17, and miR-20b) [25]. Additionally, miR-139-5p successfully arrested growth and infiltration of colorectal cancer cells by inhibiting NOTCH1 [15, 16, 22]. In the present study, we found that lncRNA RP11-59H7.3 controlled miR-139-5p-NOTCH1-mediated cell growth, proliferation, and infiltration by acting as a sponge for miRNA. To test this hypothesis, we performed luciferase reporter assays to confirm the impact of binding projected MREs on the lncRNA RP11-59H7.3 full-length transcript. As expected, the lncRNA RP11-59H7.3 reporter gene was repressed by miR-139-5p through complementary attachments. Furthermore, RNA-pull down assays further verified the role of lncRNA RP11-59H7.3 in sponging miR-139-5p to suppress NOTCH1 expression.

We also found that ectopic overexpression of lncRNA RP11-59H7.3 improved expression of NOTCH1 by sequestrating miR-139-5p. This is the first study demonstrating that lncRNA RP11-59H7.3 acts as a ceRNA to promote expression of NOTCH1 by miR-139-5p sponging, thus boosting growth, proliferation, and infiltration of CRC cells into adjacent tissues. Moreover, downregulating lncRNA RP11-59H7.3 resulted in a reduction in levels of NOTCH1, and this led to growth suppression for colorectal cancer cells.

In conclusion, the findings of this study indicate that ectopic lncRNA RP11-59H7.3 expression can act as a useful biomarker for colorectal cancer prognosis. In addition, lncRNA RP11-59H7.3 plays a role in the pathogenesis of CRC by adjusting a miRNA/targeted gene transcript transformation. Finally, our findings reveal that interfering with the lncRNA RP11-59H7.3/miR-139-5p/NOTCH1 signals could assist researchers in finding novel options for suppressing progression of colorectal cancer.

## MATERIALS AND METHODS

### Clinical samples

Fresh human specimens, paired adjacent healthy tissues and serum were obtained from colorectal cancer patients after seeking their consent between April 2017 to September 2019. After collection, the samples were immediately preserved at −80°C. A comprehensive description of the data on clinical features is provided in Table 1. No treatment was administered to any of the patients prior to surgery. Pathologists were involved in the histopathological confirmation of diagnosis of all specimens. The study was permitted by the Second Affiliated Hospital of Nanchang University (Nanchang, Jiangxi, China).

### Cell lines

We acquired NCM460, HcoEpic, LoVo, SW480, Caco-2, HCT116, HT29, and SW620 CRC cell lines, as well as HEK-293T cells from the American Type Culture Collection. The cell lines were maintained in Dulbecco's modified Eagle's medium (DMEM) [Invitrogen, USA] except LoVo cells, which were grown in RPMI 1640 [Invitrogen, USA] supplemented with 10% fetal bovine serum (FBS, Gibco, USA). All cultures were kept in a humidified incubator at 37°C and 5% CO_2_ concentration.

### RNA extraction and RT-qPCR assays

Total RNA was extracted using RNAiso Plus (Takara, Japan), then nuclear and cytoplasmic RNA purified using the PARIS^TM^ Kit (Ambion, USA). Complementary DNA (cDNA) was synthesized using the HiFiScript 1^st^ Strand cDNA Synthesis Kit (CWBIO, China), followed by quantitative real time PCR RT-qPCR using an UltraSYBR Mixture (CWBIO). Bulge-loop TM miRNA RT-qPCR primers, targeting miRNA, were synthesized by RiboBio (Guangzhou, China), and the miRNA mimics and inhibitors.

### Plasmid construction and transfection

The following constructs, sh-RP11-59H7.3 (sh-1, sh-2, sh-3) and pcDNA-RP11-59H7.3 with their respective controls (sh-NC and Vector), were designed by GenePharma (Suzhou, China) to overexpress and knock down RP11-59H7.3 in the cell lines. All shRNA sequences are outlined in Table 3. CRC cells were cultured in 6-well plates to obtain 55%–65% confluence, then transfected with the LV3 (pGLVH1/GFP/Puro) lentiviral vector (GenePharma, Suzhou, China) according to the manufacturer’s protocol.

**Table 3:** The sequences of shRNA for RP11-59H7.3.

sh-1: 5’-ATGTGAGAATGGTGTTATGGATA-3’	
sh-2: 5’-ACGAAAACGTACTTTTTAGAATA-3’	
sh-3: 5’-ATGATTGATCAGTCTACATGAAG-3’	
sh-NC: 5'-GGGCGAGGAGCTGTTCACCG-3'	
pcDNA-RP11-59H7.3:	
F: 5’-CGCGGATCCGCTTAAAAAAAAAAAGTCACTGGTG-3’ (BamHI)	
R: 5’-CCGCTCGAGCTCAGCCTCCAAAACTGCTGAAAC-3’ (XhoI)	
**The sequences for RT-qPCR**	
GAPDH	F 5’-TGTGGGCATCAATGGATTTGG-3'
	R 5’-ACACCATGTATTCCGGGTCAAT-3'
RP11-59H7.3	F 5’-TTGAGGCAAAAATGTCACTGGT-3'
	R 5’-AAAACTGGGAGAGCGAAGCA-3'
miRNA-139-5p	F 5′-ACACTCCAGCTGGGTCTACAGTGCACGTGTC-3′
	R 5′-TGGTGTCGTGGAGTCG-3′,
NOTCH1	F 5’-CGCTGACGGAGTACAAGTG-3'
	R 5’-GTAGGAGCCGACCTCGTTG-3'

A RP11-59H7.3 fragment, with miR-139-5p-binding site and the NOTCH1 3'UTR, was cloned into pLuc, while RP11-59H7.3 with the mutated seed sequence of miR-139-5p was constructed by an overlap extension PCR. The primers used in vector construction are shown in Table 3. Transfected cells were cultured on fresh culture medium, containing 4 μg/mL puromycin, to select stable puromycin-resistant cells.

### Cell proliferation and colony formation assays

We measured cell viability using the cell proliferation and viability Assay Kit (Edu, RiboBio, Guangzhou, China). Resultant colonies and cell proliferation were quantified 48h post-transfection, using 6-well plates seeded with 400 cells/well cell density. The cells were cultured for 14 days in medium supplemented with 10% FBS to allow formation of colonies, and refreshing of the medium was done every four days. Subsequently, the colonies were fixed in methanol followed, stained using 0.05% crystal violet (RiboBio) and finally manually counted.

### Analysis of cell cycle and apoptosis

Analysis of the cell cycle and apoptosis was performed on RP11-59H7.3-overexpressed and silenced colorectal cancer cells, using the Cell Cycle and Apoptosis Detection Kit (CWBIO).

### Wound healing and transwell invasion assays

We evaluated movement and invasion of CRC using wound healing and trans-well invasion assays. Briefly, colorectal cancer cells showing stable overexpression or knockdown of RP11-59H7.3 as well as their corresponding controls were briefly cultured in 6-well plates until confluence was achieved. Thereafter, they were scratched using a 10 μl pipette tip, images of cell movements taken at 0, 24, and 48h intervals, post scratching in triplicates. Cell invasion assay was then performed according to the manufacturer’s instruction of the BD BioCoat Matrigel Invasion Chambers (Becton Dickinson, Franklin Lakes, NJ). Finally, 5 random fields were counted under a light microscope.

### Western blot

Proteins were extracted using the RIPA lysis buffer (Beyotime Biotechnology, Shanghai, China) and Complete Lysis-M reagent (Roche, USA) followed by determination of their concentrations using the BCA assay (ThermoFisher Scientific, Waltham, MA). The proteins were resolved on SDS-PAGE gels (8%–10%), then transferred onto PVDF membranes for detection. We procured the following antibodies NOTCH1 (1:1000, 4380T, CST), HES1(1:1000, 11988S, CST), HEY1(1:1000, ab22614, Abcam), Bax (1:2000, ab182733, Abcam), Bcl-2(1:1000, ab32124, Abcam), E-cadherin(1:1000, 5296S, CST), Vimentin(1:1000, 5741T, CST), GAPDH (1:1000, ab9484, Abcam), from Cell Signaling Technology and Abcam (China) for use in the study.

### Luciferase reporter assay

We co-transfected HEK-293T cells with pRL-CMV, pcDNA-RP11-59H7.3, miR-139-5p mimics (Negative control, NC), and pLuc, then assayed them for luciferase activity using the Dual-Luciferase® Reporter Assay System (Beyotime, China).

### Immunoprecipitation assay

Resulting transfected cells were analyzed using the RIPA lysis buffer comprising 20 nM Tris-HCl [PH 7.5], 1% NP-40, 2.5 mM sodium pyrophosphate, 1 mM EGTA, 1 mM Na2EDTA, 1 mM Na3VO4, 150 mM NaCl, 1 μg/ml leupetin, 1% sodium deoxycholate, and 1 mM beta-glycerophosphate. Cell suspensions were centrifuged for 15 min at a 14000 rpm, and the supernatant incubated overnight, at 4 °C, with shaking prior to addition of 10 μl of beads and 2 μl AGO2 antibody. The mixture was rinsed twice using a lysis buffer and RNA extracted from the lysed cells using Trizol reagent (Invitrogen).

### *In vivo* tumor growth and metastasis assay

A total of 24 nude mice, aged between 6 and 8-weeks, were procured from Shanghai SLAC Laboratory Animal Co. Ltd for the assay. Prior to treatment, we genetically engineered SW480 cells to express a luciferase reporter gene (pcDNA3.0-luciferase), and stably transfected the resultant construct into shlncRNA RP11-59H7.3, pLVTHM, and oelncRNA RP11-59H7.3 cells (nine mices in each group). About 1×10^6^ of SW480 cells (mixed prior with Matrigel, 1:1) were carefully injected into the subrenal capsule of the mice, then a Fluorescent Imager (IVIS Spectrum, Caliper Life Sciences, Hopkinton, MA) used to monitor tumor formation and metastasis once per week. The Mice were sacrificed six weeks after treatment then tumors collected for subsequent experiments.

### Statistical analysis

Data were presented as mean ± SEM, for an average of three independent experiments. Paired *t*-tests were used for comparisons between 2 groups, with all analyses performed using SPSS software version 17.0 (SPSS Inc, Chicago, IL). Overall survival was determined using Kaplan-Meier survival curves, and the results were verified by the log-rank test. Values that showed *p < 0.05 were considered statistically significant.
